# Supplementary figures and images for: Repetitive Detection of Aromatic Hydrocarbon Contaminants with Bioluminescent Bioreporters Attached on Tapered Optical Fiber Elements
Source: Sensors (Basel). 2020 Jun 6;20(11):3237. doi: 10.3390/s20113237 (PMC7309017; doi:10.3390/s20113237)

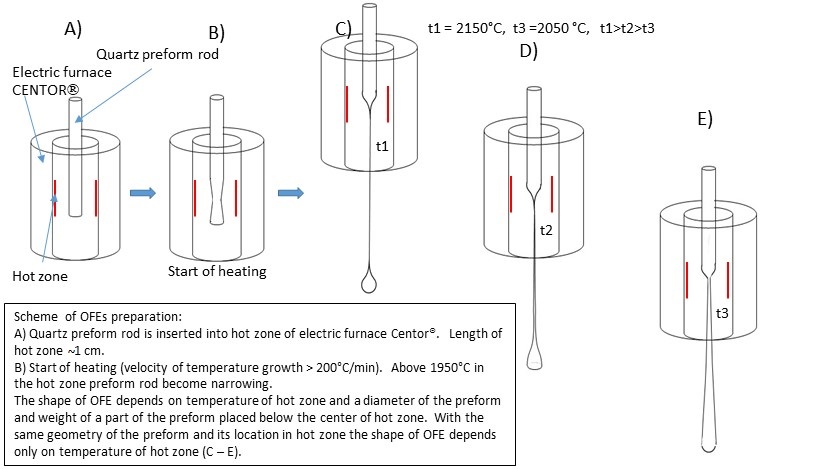

Supplement: Supplementary file 1 [file sensors-20-03237-s001.zip › Figure S1.jpg]

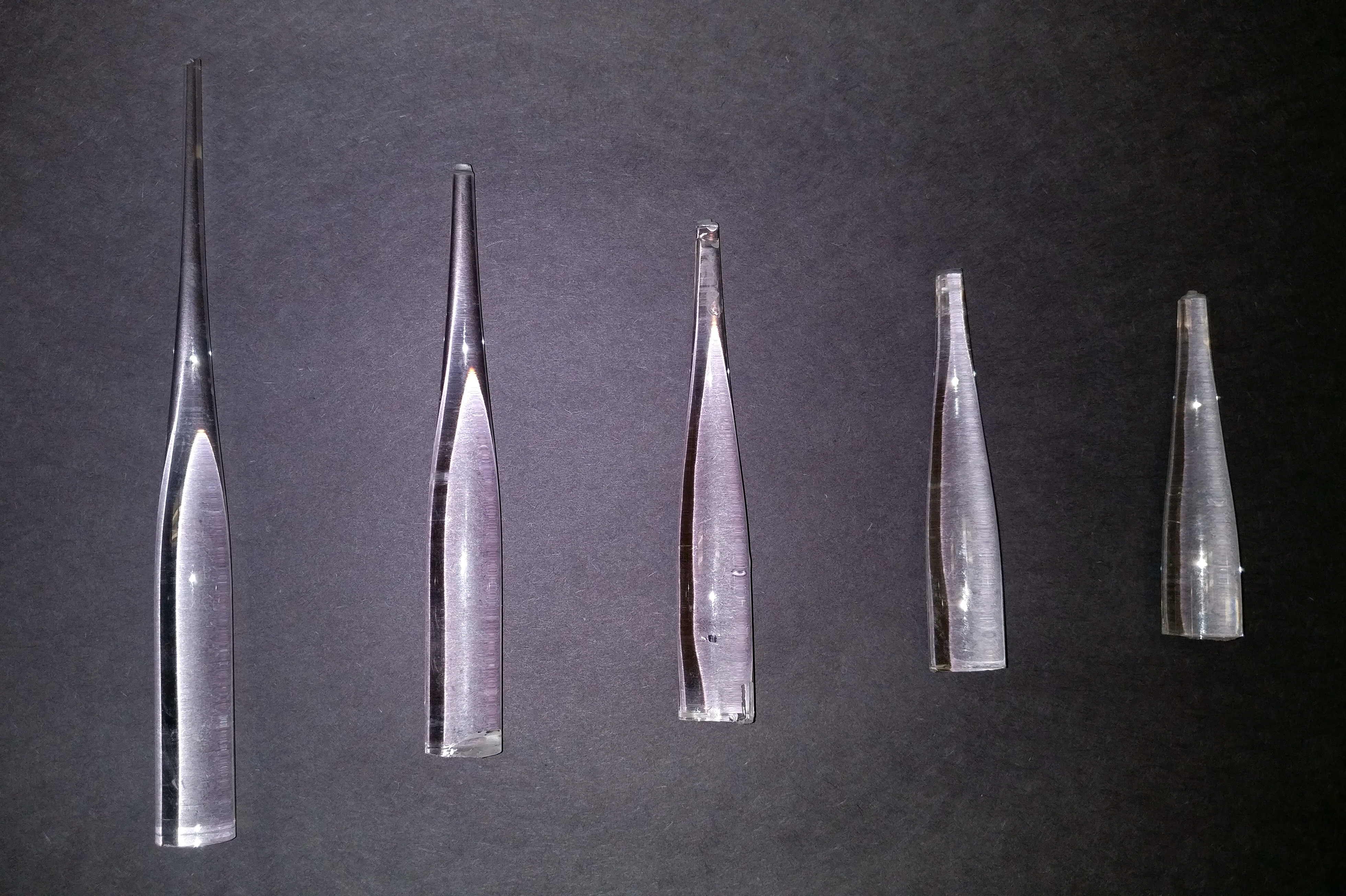

Supplement: Supplementary file 1 [file sensors-20-03237-s001.zip › Figure S2.jpg]

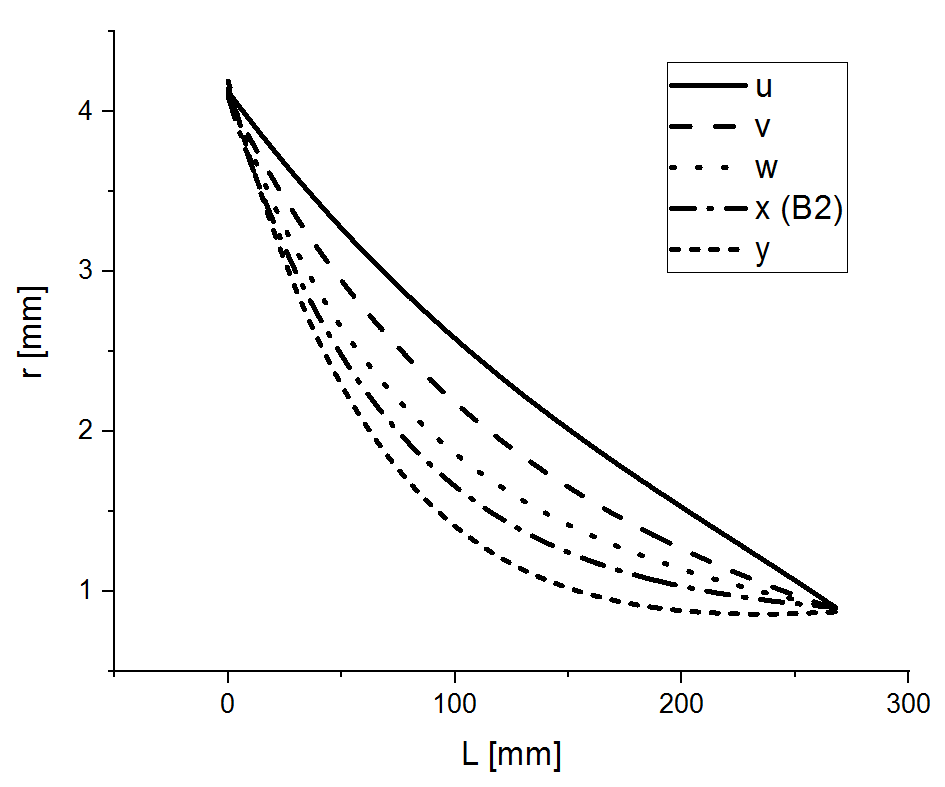

Supplement: Supplementary file 1 [file sensors-20-03237-s001.zip › Figure S3.png]

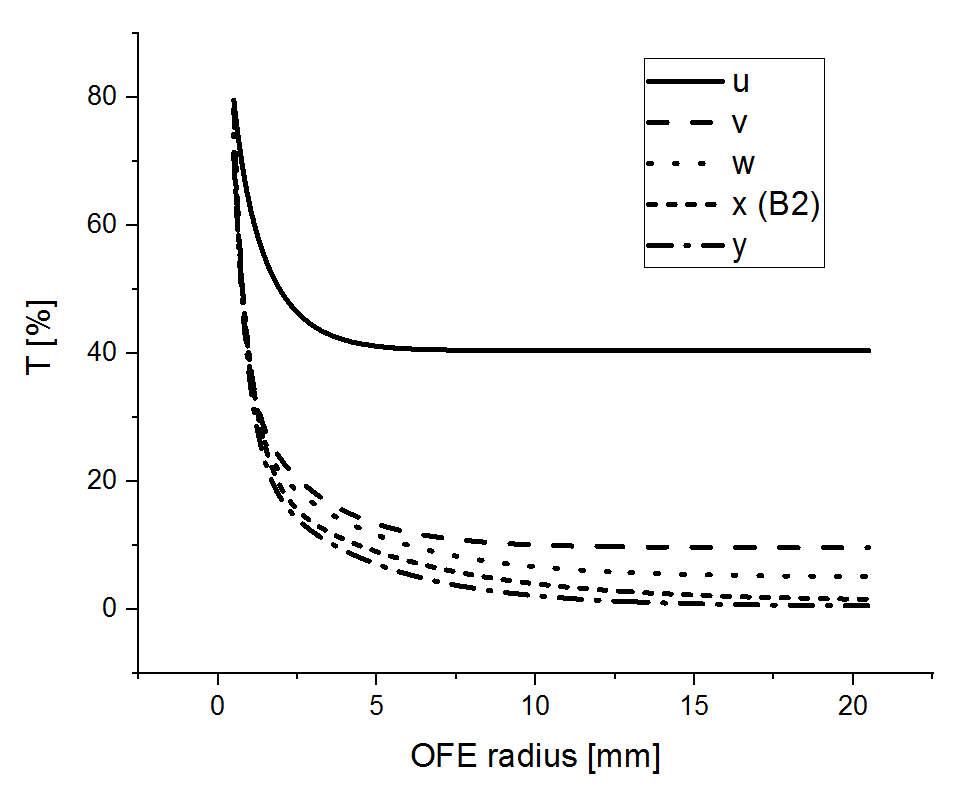

Supplement: Supplementary file 1 [file sensors-20-03237-s001.zip › Figure S4.png]

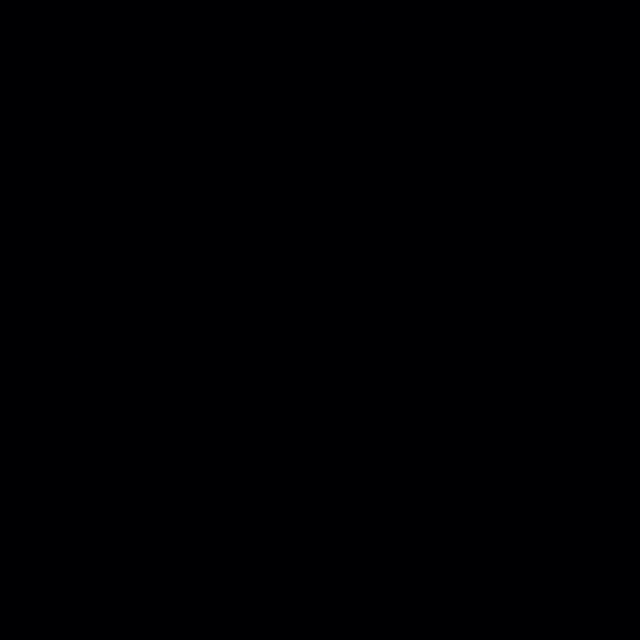

Supplement: Supplementary file 1 [file sensors-20-03237-s001.zip › Video S1.gif]
